# Chemical and Pharmacological Properties of Decoquinate: A Review of Its Pharmaceutical Potential and Future Perspectives

**DOI:** 10.3390/pharmaceutics14071383

**Published:** 2022-06-30

**Authors:** Tainá Santos Souza, Diogo Rodrigo Magalhães Moreira, Henrique Rodrigues Marcelino

**Affiliations:** 1 Graduation Program in Pharmacy, College of Pharmacy, Federal University of Bahia, Salvador 40170-115, BA, Brazil; taina.souza@ufba.br; 2 Fundação Oswaldo Cruz (Fiocruz), Instituto Gonçalo Moniz, Salvador 40296-710, BA, Brazil; 3 Department of Medicines, College of Pharmacy, Federal University of Bahia, Salvador 40170-115, BA, Brazil

**Keywords:** decoquinate, antimicrobial, physicochemical properties, medicinal chemistry, nanomedicines

## Abstract

Decoquinate (DQ) is an antimicrobial agent commonly used as a feed additive for birds for human consumption. Its use as an additive is well established, but DQ has the potential for therapy as an antimicrobial drug for veterinary treatment and its optimized derivatives and/or formulations, mainly nanoformulations, have antimicrobial activity against pathogens that infect humans. However, DQ has a high partition coefficient and low solubility in aqueous fluids, and these biopharmaceutical properties have limited its use in humans. In this review, we highlight the antimicrobial activity and pharmacokinetic properties of DQ and highlight the solutions currently under investigation to overcome these drawbacks. A literature search was conducted focusing on the use of decoquinate against various infectious diseases in humans and animals. The search was conducted in several databases, including scientific and patent databases. Pharmaceutical nanotechnology and medicinal chemistry are the tools of choice to achieve human applications, and most of these applications have been able to improve the biopharmaceutical properties and pharmacokinetic profile of DQ. Based on the results presented here, DQ prototypes could be tested in clinical trials for human application in the coming years.

## 1. Introduction

Decoquinate (DQ) is a quinolone compound traditionally used in the veterinary field for both antibiotic prophylaxis and therapy. DQ is used in the breeding of food-producing animals in combination with other antimicrobial agents [1]. Its primary use as a veterinary drug is due to its physicochemical properties, particularly lipophilicity, which hamper its solubility in aqueous fluids and prevents absorption in the gastrointestinal tract. This compound has antiparasitic, coccidiostatic, and histomonostatic (blackhead disease) effects and may therefore be used as a feed additive for birds to prevent and control coccidiosis caused by the protozoan *Eimeria* spp. [2]. This genus of parasites affects a variety of animals, including cattle, sheep, goats, and horses, which can be treated with DQ. In addition, the use of DQ in dogs and cats has been reported [3,4,5].

**Figure 1:**
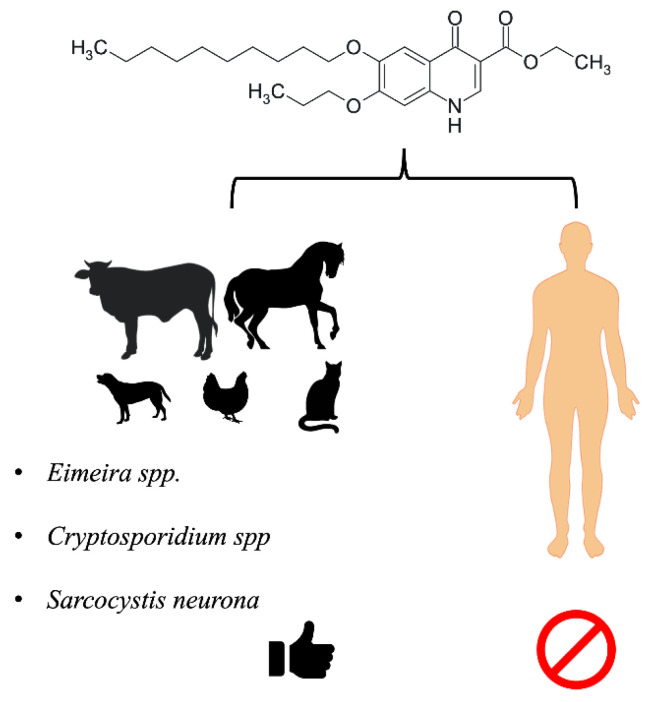
Overview of the clinical use of DQ against parasitic infections.

The similarities between *Eimeria* spp. and parasites that infect humans, as well as the ability of these microorganisms to evolve, have promoted the investigation of DQ use against other genera and species of parasites, including its potential to treat infectious diseases in humans. For example, in vitro evaluation of antiparasitic activity against *Toxoplasma gondii* and *Neospora caninum* has indicated that DQ could be used to control them. Moreover, DQ derivatives have been synthesized and shown to be active against *Mycobacterium tuberculosis*, *Schistosoma japonicum*, and apicomplexan parasites causing malaria and toxoplasmosis (Figure 1) [6,7,8,9]. In addition, DQ-loaded nanocarriers, mainly liposomes, have been developed and tested in vitro against drug-resistant *P. falciparum* and in vivo in *P. berghei*-infected mice, in vitro against *E. tenella* oocysts, and in vitro against *M. tuberculosis* [10,11,12].

The broad spectrum of DQ’s antimicrobial activity, encompassing several microbial genera, is in part due to its mechanism of action by blocking key steps in the mitochondrial electron transport system. The mitochondrial system in microbes differs from its counterpart system in mammalian cells, therefore enabling a selective activity of DQ for microbes rather than host cells. The high lipophilicity of DQ may also present potential for the accumulation and uptake of the drug in organisms, such as *M. tuberculosis*, given the presence of a bacterial cell. While the high lipophilicity of DQ is advantageous for veterinary use as a food additive to prevent absorption, this profile must be considered for potential human use as systemic antimicrobial therapy. In this review, we examine the pharmacological use of DQ in veterinary medicine, highlight the drawbacks of its use in humans, and discuss ongoing studies on the use of medicinal chemistry and nanotechnology tools to overcome these drawbacks.

## 2. History

**Figure 2:**
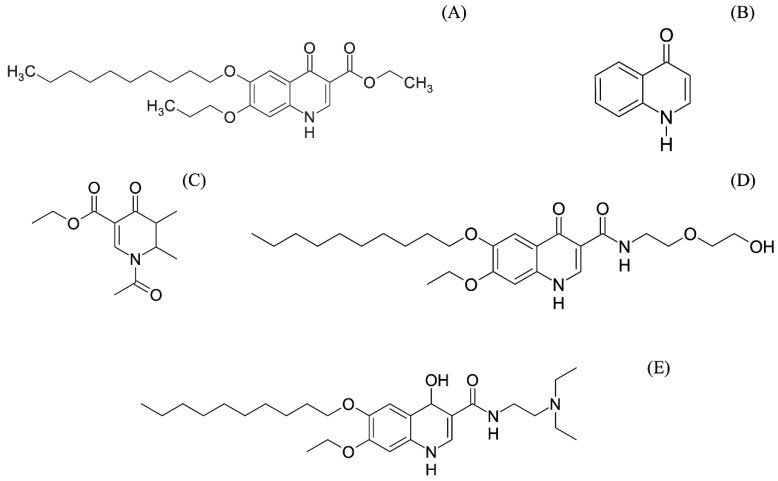
Structures of DQ derivatives with enhanced physicochemical properties for therapeutic use. (**A**) DQ, (**B**) 4-hydroxyquinoline [15], (**C**) *N*-acetyl 30 [16], (**D**) derivative RMB041 [17], (**E**) compound 15 [9].

The chemical genesis of the DQ (Figure 2A) began with the development of quinolones, a class of synthetic compounds traditionally used as antibiotics [13]. Quinolones contain in their structure the scaffold of the tautomeric 4-hydroxyquinoline (Figure 2B), which has antibiotic activity [14]. Therefore, the need to expand the therapeutic options to treat infectious diseases led to the search for derivatives with new mechanisms of action. Later, this search led to the discovery of the antimalarial activity of carboxyquinolones [15].

**Figure 3:**
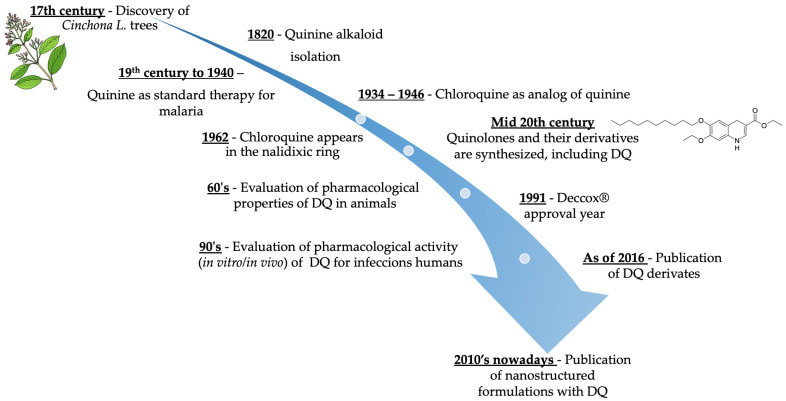
Timeline showing the history of DQ, from raw material to its approval as a drug in the present and its current applications in nanocarrier systems [14,18,19,20,22,24,25].

The chemical genesis of quinolones, such as the DQ, also had a great influence on other heterocyclic classes of compounds. For instance, it is noteworthy to cite that the first effective treatment against malaria came from quinine found in the bark of *Cinchona L*. trees (Figure 3) [18]. Elucidation of its chemical structure was established through the understanding of analogs, such as quinoline and later chloroquine. Quinine became the standard treatment for malaria in the mid-19th century until the 1940s, but with the emergence of drug-resistant strains of *P. falciparum*, its therapeutic use was curtailed, but it is still most commonly used against erythrocyte schizonticides for noncomplicated malaria [19,20,21]. Its chemical structure was well established by French chemists in 1820 through the understanding of analogs, such as quinoline and later chloroquine [22,23]. Curiously, an impurity in the synthesis of chloroquine led to the detection of nalidixic acid, the first antibacterial quinolone derivative [24].

## 3. Physicochemical Properties

Under normal temperature and pressure conditions, DQ is a crystalline powder with an off-white color. It has a mild odor and a melting point between 242 and 245 °C. Its molecular weight is 417.6 g/mol. It has an octanol/water partition coefficient ≥5.7 at pH 5, 7, and 9, solubility in water of 0.06 mg/L (20 °C), and in aqueous buffer at pH 4.9 of 0.01 mg/L. The low vapor pressure (3 × 10^−9^; 25 °C) indicates that this substance does not volatilize at high temperatures [26]. Despite its beneficial antimicrobial activity against parasitic diseases, DQ is poorly soluble in water and is rapidly metabolized. This drug profile is partly due to a combination of chemical parameters, such as the presence of an ethyl ester group, a carboxylic acid group, and a labile hydrogen in the *N*-1 of the quinolone ring. These chemical groups are prone to a high rate of metabolization. Nowadays, these drawbacks can be overcome by pharmaceutical technology and medicinal chemistry approaches [15].

## 4. Pharmacological Use

A large number of DQ derivatives have been synthesized over the years. An important example of derivatization of DQ towards drug optimization was achieved when the ethyl ester group was replaced by amides, and the H atom in the *N*-1 position of the quinolone heterocycle was replaced by alkyl groups, increasing the polarity of the resulting molecules. Another example is the *N*-acetyl derivative designated 30 (Figure 2C), which showed activity five times higher than DQ against chloroquine-sensitive *P. falciparum*. The ratio between the IC_50_ for lung fibroblast cell line WI -38 and the IC_50_ for *P. falciparum* NF54 showed that this derivative is more selective than DQ (selectivity index equal to 20, 408 and 3759, respectively). Thus, conversion of the DQ ethyl ester into more polar alcoholic esters and less easily metabolized amides is a possible way to solve the physicochemical problems of the molecule. Further studies are needed to clarify whether the improved efficacy of the derivatives can be translated into molecules of therapeutical efficacy in experimental infection models in rodents and humans [16,27].

Although the use of DQ is limited because of problems related to its physicochemical properties, several preclinical studies have investigated treatment with DQ in experimental animal models against microbial infections, including treatment with DQ alone or in combination with other drugs. Drug combinations of DQ and other antibiotics such as bacitracin and lincomycin, tylosin, monensin, or chlortetracycline have been described against bacterial enteritis and bacterial pneumonia [1]. Most importantly, Fry and Williams found synergism of antiprotozoal activity between DQ and clopidol, another coccidiostat agent, both in vitro and in vivo, potentiating this effect and inhibiting electron transport in *E. tenella* [28,29]. This mechanism was first observed in 1975 when it was discovered that coccidiostatic quinolones likely inhibit mitochondrial cytochrome *b* of non-sporulated oocysts of *E. tenella* [30]. These studies led to the hypothesis that the mechanism of action of DQ is via inhibition of mitochondrial oxygen consumption, which was later confirmed by a high-throughput screen in retinal ganglion cells (RGC-5) [31]. Therefore, it is likely that DQ prevents the development of sporozoites after they have invaded the intestinal epithelium by inhibiting parasite mitochondrial respiration [32].

### 4.1. Veterinary Use

DQ is the active ingredient in Deccox^®^, a drug added to animal feed and used mainly to prevent coccidiosis in birds, goats, cattle, and sheep. DQ is approved as a synthetic compound in the European Union, USA, New Zealand, Japan, Korea, and China as a zootechnical additive at a common dose of 20 to 40 mg/kg feed. Typically, 500 g per ton of feed is added, resulting in 30 mg/kg of DQ [2,6,33].

For birds, especially chickens raised for human consumption, the most economically important coccidial parasites are *E. acervulina*, *E. maxima*, and *E. tenella*. These pathogens alter the microbial environment of the intestinal tract, disrupt the digestive process, and cause severe intestinal damage that can lead to the death of the animal [34,35]. The best control measure is prophylaxis, and for this purpose, DQ is widely used [36]. To verify this information, an unprecedented field study on farms in the UK can be highlighted. It showed that no clinical coccidiosis occurred when DQ was administered to chickens. Furthermore, no adverse effects or side effects were observed in the animals [25].

In another study involving farms in Minnesota, USA, dairy farms were evaluated for six months for prevalent dietary risk factors associated with fecal shedding of Shiga-toxin-coding bacteria (STB). An increase from 1.6 to 4.5% positive STB cases was observed in weaned calves without the use of Deccox^®^, especially in small herds. DQ would then have a protective effect against STB in these animals [37].

In addition, several studies report the activity of DQ against various parasites that cause common infections in the veterinary clinic (Table 1). Compelling evidence suggests that DQ can be used to prevent the recurrence of hepatozoonosis in dogs by preventing reinfection by *Hepatozoon americanum*. This parasite causes hepatozoonosis in dogs, and its infection was investigated in a retrospective study of 53 dogs to evaluate the clinical and pathological findings before and after treatment. The animals were divided into two groups, a short-term and a long-term group. In the latter, 22 of the 27 dogs survived for 21 months, with 11 dogs no longer showing clinical signs while still receiving DQ, suggesting that prolonged administration resulted in better survival of these animals [38,39].

Moreover, administration of DQ also reduced oocysts and clinical signs of cryptosporidiosis caused by *Cryptosporidium* spp. Lallemond and coworkers showed that calves treated orally with 2.5 mg/kg daily for 28 days had a higher average daily weight gain [40]. Mancassola et al. also orally inoculated 20 ruminants with 106 *Cryptosporidium parvum* on the first day of life. Later, they were divided into groups: Group A, which received no treatment (control), and Group B, which received 2.5 mg/kg DQ daily for 21 days from the third day of life. After inoculation, both groups had diarrhea and high excretion of oocysts. However, group B had no mortality, a lower number of diarrhea events, and lower excretion of oocysts [41]. In a field study of suckling calves and their lambs, a decrease in cryptosporidiosis was achieved when DQ was administered four weeks before birth and one week after birth [5].

In another study on the treatment of cryptosporidiosis, Ferre and coworkers studied goats with neonatal diarrhea caused by *Cryptosporidium* spp. for 21 days. A total of 64 juvenile goats (kids) and 14 pregnant goats were divided into three groups: A, the control group with 24 goats receiving a placebo; B, consisting of 25 goats receiving 2.5 mg/kg per day of orally administered DQ in two doses; and group C, consisting of 15 goats and 14 goats receiving 2.5 mg/kg of orally administered DQ once daily. As a result, the number of goats in group A that suppressed *Cryptosporidium* spp. was greater than that in group B, as were the goats in group C, including neonates. Group C had the best weight gain. DQ reduced the number of diarrheal episodes, improved fecal consistency, and delayed the onset of clinical cryptosporidiosis without increasing mortality. Therefore, the use of DQ as a prophylactic treatment was suggested because the results in group C were similar to those in group B and better than those in untreated children (group A) [42].

DQ was also tested against besnoitiosis, a disease caused by the protozoan *Besnoitia besnoiti*, which is similar to *Toxoplasma gondii* and affects cattle, horses, donkeys, and goats. The parasite was grown in vitro in monkey embryo adrenal macrophage cells (MARC-145) and exposed to various compounds. The DQ showed an IC_50_ of 10 nM against the parasite, indicating good safety and efficacy values. The cytotoxicity tests confirmed that the DQ does not act on the host cells but only on the parasite. As predicted, the DQ acts mainly on the mitochondria of the parasite, which was confirmed by transmission electron microscopy [43].

Additionally, in the treatment of merozoites of *Sarcocystis neurona*, a protozoan that causes the neurological disease myeloencephalitis caused by equine protozoans, treatment with DQ completely arrested the growth of merozoites within 10 days. In this assay, DQ was used at a concentration of 240 nM [6]. In addition, the drug showed excellent in vitro activity against *Neospora caninum*, a coccidian protozoan that infects dogs and cattle and is a major cause of abortions in dairy cows, with significant veterinary and economic implications. The DQ acted rapidly against the intracellular stages of *Neospora caninum* tachyzoites, causing their death after 5 min at a concentration of 0.1 µg mL^−1^ in cell cultures [4,38]. As in pregnant calves infected with *N. caninum*, a dose of 2 mg/kg orally reduced abortions and associated neonatal infections by the eighth month of gestation [44].

### 4.2. Human Use

Considering that DQ has a unique structural feature and broad-spectrum antimicrobial profile for use in veterinary medicine, its potential for treating microbial infections in humans is attractive and has been studied against several pathogens in recent years. A summary of these in vitro studies is provided in Table 1. Below, we review in more detail the pharmacology and mechanism of action studies of DQ and discuss the drawbacks associated with its low solubility and how these can be addressed and contribute to the development and therapeutic innovation in infectious diseases.

#### 4.2.1. Malaria

Current malaria therapy is undermined by the continued emergence of parasite resistance [45], lack of therapeutic options for certain species (such as *P. vivax*) [46], and treatment of multiple (hepatic and sexual) stages other than the asexual blood stages (i.e., ring, trophozoite, schizont) [47,48]. In the search for the next generation of drugs to prevent the spread of the disease, a new potential drug candidate should have antiplasmodial activity for multiple stages of the *Plasmodium* life cycle, efficacy in experimental models, and a known mechanism of action. Several research groups have demonstrated the in vitro potency and in vivo efficacy of DQ against the asexual blood stages (blood schizonticidal) and hepatic stages (tissue schizonticidal) of *Plasmodium* [49,50,51,52].

**Table 1:** (A) Common DQ dose regimen in clinical research or models evaluating it in animal cell cultures. (B) In vitro activity of DQ and its derivatives or models evaluating it against parasites infecting humans.

	*Etiological Agent*	Dose	Parameter	Assay	Reference
*A*	*Hepatozoon americanum*	10 to 20 mg/kg	-	Clinical *	[39]
	*Cryptosporidium* spp.	2.5 mg/kg	-	Clinical *	[40,41,42]
	*Besnoitia besnoiti*	0.24 nM to 240 nM	IC50 10 nM	In vitro	[43]
	*Sarcocystis neurona*	240 nM	IC50 0.5 nM	In vitro	[38]
	*Neospora caninum*	2 mg/kg	0.1 µg mL^−1^	In vitro	[4]
*B*	*Plasmodium berghei (liver stage)* *Mycobacterium tuberculosis* *Toxoplasma gondii* *Plasmodium yoelii* *Schistosoma japonicum*	10 mg/kg 20 mg/kg - 50 mg/kg 10 µmol/L	IC50 = 2.6 nM MIC90 = 1.61 μM IC50 = 0.005 µg/mL IC50 = 177 pM -	In vitro In vitro In vitro In vitro In vitro	[7] [17] [8] [51] [9]

* Animal tests for veterinary drugs.

In 2012, a more complete antimalarial profile was established for DQ. In this study, da Cruz et al. performed an extensive drug screening against the malaria hepatic stages using the model of *P. berghei* sporozoite infection in Huh7 liver cells. DQ showed not only a potent in vitro antimalarial activity but also an oral efficacy in the experimental model of liver infection in mice. The potency and efficacy of DQ were greater than primaquine, a reference tissue schizonticidal drug [7]. While another reference drug for hepatic stages, atovaquone, has no antimalarial activity for gametocytes—the parasite stage responsible for human-to-insect transmission—DQ was able to reduce gametocyte viability. This suggests that DQ has a broad antimalarial activity for multiple stages of the *Plasmodium* life cycle.

**Figure 4:**
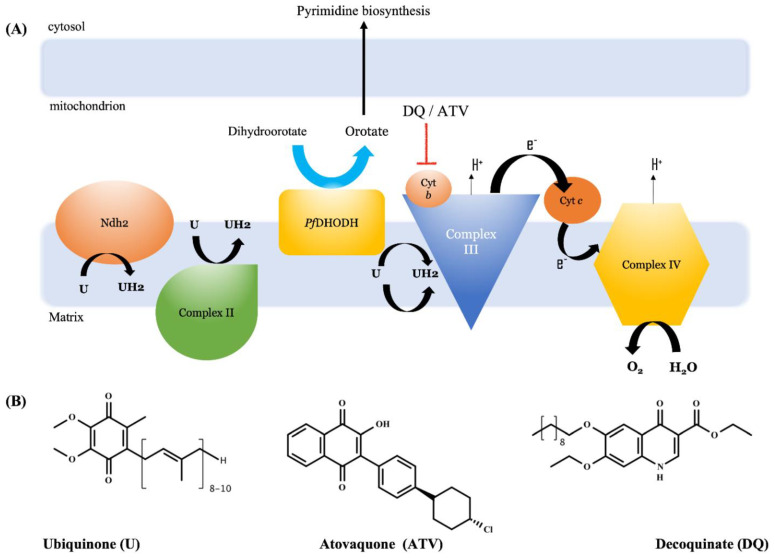
Overview of pyrimidine metabolism in *Plasmodium* parasite and its drug blockage. Panel (**A**) shows that ubiquinone is recycled by redox chemistry from the dehydrogenase enzymes’ rotenone-insensitive internal NADH dehydrogenase (Ndh2) in complex I and *P. falciparum* dihydroorotate dehydrogenase (*Pf*DHODH) in complex II. Complex III is involved in pyrimidine biosynthesis and regulation of the complex IV, which is necessary for the electron transport chain. Drugs that bind to the ubiquinone-binding site of cytochrome *b* of the *bc_1_* cytochrome complex block the recovery of ubiquinone by *Pf*DHODH and the transfer of electrons to downstream acceptors in the complex IV. This leads to a breakdown of the mitochondrial membrane potential and the subsequent death of the parasite. Panel (**B**) shows the chemical structures of ubiquinone (U) and the antiplasmodic drugs atovaquone and decoquinate, which compete with ubiquinone for the binding site in cytochrome *b* (Cyt b).

The cytochrome *bc_1_* complex is essential for ubiquinol recycling and electron transport chain homeostasis in mitochondria, while the plasmodium enzyme dihydroorotate dehydrogenase (*Pf*DHODH) is required for pyrimidine biosynthesis from the precursor orotate. Its blockage is therefore lethal to the parasite (Figure 4) [53,54,55,56]. Regarding the antimalarial mechanism of action of DQ, da Cruz et al. [7] demonstrated with convincing experiments in cell cultures and in isolated mitochondrial particles that DQ, like atovaquone, achieves highly potent antimalarial activity by binding and subsequently blocking the cytochrome *bc_1_* complex of plasmodia. Using cell culture assays, these authors showed that DQ does not inhibit the enzymatic activity of *Pf*DHODH or the cytochrome *bc_1_* complex in human embryonic kidney cells (HEK293).

Other independent research groups have also demonstrated the potency and efficacy of DQ in the treatment of malaria, especially as a tissue schizonticidal agent. For instance, DQ was observed to provide partial protection when administered to mice infected with *P. yoelii* at a single oral dose of 50 mg/kg [51].

It is important to highlight that atovaquone, a potent broad-spectrum antimalarial agent, is already used in patients as a drug combination with proguanil (Malarone^®^), which exerts its effects via the same mechanism of action as DQ. However, atovaquone presents several challenges that warrant the development of DQ and derivatives for malaria therapy. First, parasites can rapidly acquire resistance to atovaquone. Da Cruz et al. [7] and Nam et al. [51] have shown that parasites presenting resistance to atovaquone are susceptible to treatment with DQ, indicating a limited cross-resistance between DQ and atovaquone. Second, atovaquone has limited toxicity against mature gametocytes, although it has a strong effect on other sexual stages in the insect vector [57]. DQ has a more consistent activity in reducing gametocyte viability. Thus, DQ has a potentially advantageous drug profile compared to atovaquone. Additionally, the efficacy of DQ in experimental rodent malaria models suggests that a high dosage is required to completely suppress infection compared to atovaquone [51]. This problem is likely related to the low hydrophilicity of DQ and the resulting biopharmaceutical and pharmacokinetic properties. Regarding the problem of low hydrophilicity of DQ, several research groups have found that its relatively low efficacy as a schizonticidal drug can be solved by nanotechnology-based pharmaceutical formulations [49,50,51,52]. However, it remains unclear whether the in vitro activity of DQ against gametocytes can be converted into an effective therapy to block transmission, as no study of this efficacy has yet been conducted in suitable models of malaria transmission exposed to DQ.

#### 4.2.2. Tuberculosis

Tuberculosis (TB) is an infectious disease caused by the etiologic agent *Mycobacterium tuberculosis* (Mtb). Antimicrobial therapy to control TB faces several challenges: (i) drug permeation through the cell wall of Mtb, (ii) drug biodistribution (local and systemic), long-term therapy (usually six months) that affects patient adherence and compliance, and (iii) the spread of drug-resistant strains to current therapy [58].

Quinolones have been used in the clinic to treat a variety of infectious diseases of the lung since the discovery of the first antibiotic quinolone, nalidixic acid. For this reason, DQ has the potential as an antimicrobial agent against Mtb [59]. In in vitro tests against Mtb H37Rv, DQ is virtually inactive and has a MIC > 125 μM [27]. Nevertheless, efforts have been made in both pharmaceutical and medicinal chemistry to utilize DQ for TB therapy.

In a medicinal chemistry effort, Beteck et al. pursued the derivatization of DQ, which led to the identification of compounds exhibiting antimicrobial activity against Mtb with 5- to 10-fold higher potency than DQ. Based on a pharmacophore model constructed using structure–activity relationship data, the authors concluded that the lack of activity of DQ is likely due to its limited ability to penetrate the cell wall of Mtb, whereas its derivatives can penetrate the cell wall more effectively [27]. Subsequent studies to understand the antimycobacterial activity of DQ investigated the mechanism of action, pharmacokinetics, and biodistribution of a DQ derivative called RMB041 (Figure 2D). The metabolomic signature of Mtb H37Rv ATCC 27,294 exposed to RMB041 showed significant changes in the levels of metabolites associated with the cell wall, suggesting that RMB041 achieves its antimycobacterial activity by interfering with the homeostasis of the Mtb cell wall [60]. In another study, the RMB041 molecule showed high solubility (9.99 g/L), in contrast to DQ itself, which has low solubility, reducing its gastrointestinal absorption and limiting its therapeutic use [17]. Moreover, new compounds from DQ have been observed to increase oral bioavailability by up to 20% in mice. The in vivo half-life (t_1/2_) of the derivative was also found to be medium to long (150 min), which would reduce the cost of current therapies and improve treatment adherence by favoring the shortening and duration of TB treatment [17,61,62]. To date, it is unclear whether the in vitro activity of DQ derivatives against Mtb can be translated into effective therapy for TB, as no systematic study of efficacy has been conducted in murine models of TB infection.

In a formulation effort, topical nanoemulsions containing DQ were also used to treat TB. These systems were effective in releasing the drug in the stratum corneum–epidermis and epidermis–dermis. They were not cytotoxic to the immortalized human keratinocyte cell line (HaCaT) and inhibited Mtb in vitro. These results were primarily obtained with the formulation containing DQ and olive oil, which achieved an inhibition level of 54% against Mtb H37Rv, compared to the formulation containing DQ, clofazimine, artemisone, and olive oil, which achieved 53%, suggesting that these topical nanoemulsions successfully deliver the active agent both individually and in combination in the topical treatment of cutaneous tuberculosis in a complementary manner [63].

#### 4.2.3. Toxoplasmosis

Assessing the resistance of *Toxoplasma gondii* to some anticoccidials, studies found that cytochrome mutations are related to the parasite’s resistance to DQ, so this would be the target to achieve the respiratory chain inhibition mechanism of mitochondrial electron transport [64]. DQ and other anticoccidial drugs were evaluated for activity against *Toxoplasma gondii* in human fibroblast cultures. Of the 13 drugs, 9 showed selective antitoxoplasma activity, with DQ being the second most effective with an IC_50_ = 0.005 µg/mL. It was surpassed only by monensin (IC_50_ 0.001 µg/mL) [8].

Therefore, the efficacy of DQ against experimental toxoplasmosis was tested in pregnant sheep that received the drug orally from 90 days of gestation to parturition. At a dose of 2 mg/kg, DQ delayed the onset of the febrile response to *T. gondii* infection and reduced the overall severity of fever and placental damage caused by the parasite. It also prolonged the average gestation period and increased the number and weight of lambs born compared with sheep that did not receive DQ [65].

#### 4.2.4. Schistosomiasis

The activity of DQ derivatives against *Schistosoma japonicum* was also discovered. Among them, the so-called “compound 15” (Figure 2E) synthesized by Wang and coworkers with two diethylamino groups eliminated 100% of adult *S. japonicumin* in only 72 h at a concentration of 10 µmol/L. This result suggests that it could serve as a promising lead compound for the development of new drugs against schistosomiasis [9].

### 4.3. Resistance to DQ Therapy

One of the challenges in developing new antimicrobial and antiparasitic agents is the possibility of cross-resistance to existing therapies. Another challenge is that some biochemical processes that are targets of drugs are more susceptible to mutation, resulting in microbes with lower sensitivity to drugs. The most studied microbe to date in acquiring resistance to DQ therapy is *Plasmodium*. Resistance to drug therapies has been documented for almost all antimalarials on the market today. To prevent the development of parasite resistance, therapeutic management with drugs that inhibit the major pathogens is required. In the case of cytochrome *bc_1_* inhibitors, new antimicrobial agents are being explored primarily with this goal in mind [66,67]. DQ has previously been described as a drug that exhibits cross-resistance. Nanocarriers are among the options that have been explored in recent years to combat drug resistance [68].

#### 4.3.1. Malaria

A potential limitation of DQ, which is actually observed across different classes of molecules that inhibit the mitochondrial electron transport chain in microbes, is the mutation in the binding site of the target and the subsequent development of drug-resistant parasites. Second, drugs targeting the mitochondrial electron transport chain in *Plasmodium* often exhibit a relatively slow antiparasitic activity compared with other antimalarial drugs [69].

It should be clarified that DQ has limited cross-resistance to atovaquone-resistant parasites, with mutations A122T and Y126C at the cytochrome *b* binding site. However, one mutation that occurs in atovaquone is Y268S, which leads to late treatment failure [66]. Another mutation that leads to atovaquone resistance and corresponds to one of the most common mutations in *P. falciparum* is Y279S, which led to the reversal of the DQ snapping position. Therefore, DQ is expected to effectively inhibit the cytochrome *bc_1_* mutant Y279S with little cross-resistance with atovaquone. These inhibitors have different binding modes, as shown by molecular docking studies [7,51].

Corey and coworkers, using 50 antimalarials identified by phenotypic screens, found that fast-acting compounds prevented the development of resistance and concluded that these compounds rapidly halt disease progression and prevent severe complications. Thus, when analyzing resistant mutants, early resistance may not be a major barrier to new malaria candidates [70]. However, in the case of DQ, it is still unclear whether it has a fast-acting antimalarial property to inhibit parasite growth.

#### 4.3.2. Toxoplasmosis

In an effort to understand the mechanisms of drug resistance and the sites of action in parasites, Montazeri and co-authors investigated drug resistance in *T. gondii*. It was found that mutant *T. gondii* parasites infected with 200 μg/mL ethylnitrosourea became 20-fold less susceptible to DQ in fibroblasts infected with 4 × 10^7^ of these mutant *T. gondii* [8,71]. Similarly, the ethylnitrosourea mutant DeqR-1 was 1000-fold more resistant to DQ compared to the wild parasite (clone). In contrast, it was more sensitive to atovaquone. The mutation that confers resistance to DQ alters a mitochondrial component, possibly making the mutant more sensitive to inhibition by atovaquone [72]. One of the challenges of antimalarial resistance is that it has been documented for almost all antimalarials on the market today. Careful therapy against the development of parasite resistance involves therapeutic management with drugs that inhibit the major pathogens. In the case of cytochrome *bc_1_* inhibitors, new antimicrobial agents are being explored primarily with this goal in mind [66,67]. DQ, for example, has already been described as a drug that exhibits cross-resistance, i.e., the ability of coccidia to survive the anticoccidial effects of a drug to which they have never been exposed because they have already developed resistance to a chemical agent [73]. Options that have been explored in recent years to combat drug resistance include nanosystems [68].

## 5. Biopharmaceutics and Pharmacokinetics

The biopharmaceutical properties are solubility and permeability. It has always been known that these properties are important regardless of the route of administration. However, they received more attention in the 1990s when Amidon and his collaborators created the Biopharmaceutical Classification System (BCS) for orally administered drugs [74]. The BCS has four main classes, with BCS class II divided according to the character of the molecule (weak acid or weak base) [75]. The classification is based on the solubility of the drug in aqueous fluids (water, simulated gastric fluid, and simulated intestinal fluid) and permeation in the gastrointestinal tract.

As described in Section 3, DQ is known for its poor water solubility, and there are no permeability studies because it is impossible to perform the tests in aqueous media. This is likely the reason for the use of soybean oil and lecithin in the Deccox^®^ formulation. Nevertheless, Van Zyl and coworkers [12] (2019) recently performed a skin permeation test with DQ suspensions and DQ-loaded liposomes, niosomes, and transferosomes. The DQ suspension was not able to penetrate the skin. Among the nanocarriers, niosomes and transferosomes were the most efficient. Niosomes induced the highest concentration of DQ in the stratum corneum–epidermis, 1.54 μg/mL, compared with 1.32 μg/mL for transferosomes. Other nanotechnological approaches to enhance DQ absorption were also performed, but they directly investigated absorption (pharmacokinetics) rather than permeation. Therefore, they are described later in this section.

The pharmacokinetics of DQ in humans are still unknown, but pharmacokinetic parameters have been described for some healthy animals. In chickens, DQ is absorbed in the gastrointestinal tract. In addition, DQ metabolites can be detected in the kidneys and liver, and excretion occurs in the urine. This metabolic pathway has also been observed in rats, cows, and sheep. In addition, there are no relevant interactions with other food additives or veterinary drugs, except bentonite, a clay mixture used as a compaction and dispersion agent, which affects testing, but not DQ activity [76].

Initial pharmacokinetic studies in chickens show low accumulation of residues in tissues, with the highest DQ concentrations found in the liver and kidneys, in contrast to muscle, where low concentrations occur, but residues continue to be found in skin/fat. After the end of dosing, DQ was rapidly excreted, and no detectable residues (0–1 ppm) were found in any tissue at 34 days after dosing [77,78].

The most recent studies on in vitro and in vivo pharmacokinetics describe the pharmacokinetic parameters of DQ assays and formulations to improve biopharmaceutical properties, including nanotechnology and medicinal chemistry approaches. The structures studied included micro- and nanosuspensions.

Wang’s work addressed the preparation of dispersions and particle size reduction through sonication and high-pressure homogenization, creating a more suitable dosage form to facilitate absorption and improve the stability of DQ. Both micro-suspensions and nanosuspensions were prepared, but the nanosuspension was better absorbed than the micrometric suspensions. The nanosuspension had an average size of about 0.4 µm, and its use at a single oral dose of 80 mg/kg in mice resulted in a 14-fold increase in area under the curve (AUC) and a 4-fold increase in maximum concentration (C_max_) compared to the suspension in microparticles, where the average size was about 36.88 µm (Table 2) [79]. This formulation consisted of surfactants, including Tween^®^ 80, and these components tended to improve the biocompatibility of the materials and increase permeability due to the interactions of their groups with lipid bilayers, in addition to increasing the bioavailability of lipophilic drugs in the gastrointestinal tract [80].

In a blood phase study of malaria infection with intramuscularly administered DQ, solid dispersions were prepared using peanut oil as an oily vehicle, and the particle size of this formulation was also reduced by sonication and high-pressure homogenization. It was found that the prepared micro-suspension exhibited the slowest release at the injection site and prophylactic activity in a mouse model infected with *P. berghei* sporozoites over eight weeks post-infection (Table 2). The t_1/2_ elimination in mice treated with the micro-suspension (8.31 μm) was 1.92-fold greater than in animals treated with the nanosuspension (0.43 μm), and the AUC of the micro-suspension after a single dose of 120 mg/kg was 1.76-fold greater than the AUC of the nanosuspension. Intrinsic clearance rate data calculated in treated animals showed that the nanosuspension was released almost twice as fast as the micro-suspension. This release indicates that the microparticles remain in a large depot at the injection site, suggesting that a new method can be provided for long-term prophylaxis by proposing a slow-release formulation with increased drug exposure time [49].

To evaluate the absorption, distribution, metabolism, and excretion in vitro (Table 3), some parameters were calculated according to Tanner and coworkers. The results showed that they were low for RMB733 and RMB041 derivatives and high for RMB043. The oral bioavailability in mice was 20% for RMB041 and RMB043 and 5% for RMB733. These properties of long, flexible alkyl chains that allow the compounds to penetrate the cell membranes and mycolic cell wall of Mtb, as well as their relatively inexpensive production cost (USD 10/kg) involved, make these new compounds potential agents for TB therapy [16,17]. Moreover, the in vitro t_1/2_ determined by microsomal degradation rates was relatively long and, together with a high volume of distribution values, represents an important feature for the development of agents capable of penetrating the parasite granuloma [81,82]. An analysis of hepatic clearance can also help determine whether the primary clearance pathway is metabolic or whether the drug is excreted unchanged [17].

## 6. Toxicity

The toxicity of DQ has been analyzed from different aspects, mainly (i) toxicity to animals, (ii) toxicity to humans, and (iii) environmental toxicity [2,26].

Toxicity to animals is the most important aspect because DQ is already used to treat various animal species. However, the toxicity studies were mainly conducted on chickens and rats. No significant biochemical or hematological risk was found in the various evaluations performed in chickens—although differences were found in the same tests—including a study that evaluated the microbiota of the gastrointestinal tract of chickens and found that bacteria were not affected at concentrations much higher (>64 mg/L) than those expected in this region. Therefore, DQ was considered safe at the therapeutic concentration of 40 mg/Kg [2].

Regarding the continuous use of DQ, studies were conducted in rats and dogs over a period of 16 days to 2 years. In rats, the lowest no observed effect level (NOEL) was 15 mg/Kg in dogs. No adverse effects were observed in rats over a 2-year period at doses up to 37 mg/Kg. In addition, no carcinogenic, embryotoxic, teratogenic, or generational (up to three generations) toxicity was observed in rats. However, fetotoxicity was observed at 300 mg/Kg, NOEL at 100 mg/Kg.

Toxicity to humans is not measured directly from human exposure to chemicals but indirectly from animal studies. For example, the safety of DQ for users has been evaluated in inhalation studies where no irritation was observed. Skin and eye irritation studies also revealed no sensitization. In addition, toxicity associated with indirect human consumption of DQ was considered unnecessary due to the rapid metabolism of DQ in animal organisms, mainly chickens [2,79,83].

The third consideration for the toxicity of DQ is the environmental conditions, which were evaluated for terrestrial, aquatic, and sediment experimental conditions. All analyzes have shown that decoquinate—even at the highest proposed doses—is safe for use in chickens and poses no risk of secondary poisoning or groundwater contamination [2].

Therefore, the use of DQ is considered safe to this day. However, toxicological evaluation and observation of side effects and interactions must be considered when developing new DQ formulations.

## 7. Patents

A search of the ESPACENET database conducted through July 2021 classified patents containing DQ according to the codes provided by the World Intellectual Property Organization (WIPO) in International Patent Classification (IPC) and Cooperative Patent Classification (CPC) systems for classifying applications.

**Figure 5:**
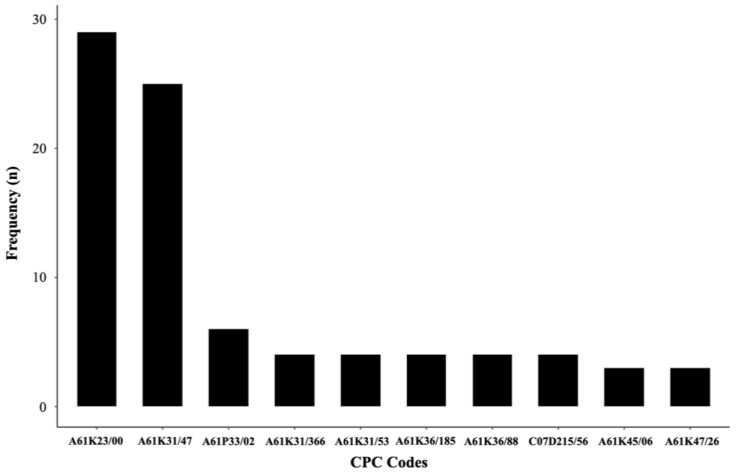
Distribution of CPC codes in DQ patents available in the ESPACENET database.

CPC A61K2300/00 and A61P33/02 are the most commonly used (Figure 5). The first code refers to antiparasitic and antiprotozoal agents, and the second to combinations of active ingredients. Code A61K31/47, on the other hand, is classified in both IPC and CPC and refers to isoquinolines and quinolones, a class to which this drug also belongs. It is also noted that patents for this drug are mainly from China and the United States and are primarily filed by industry compared to universities and research institutes.

Some inventions established the use of traditional DQ as an additive for use against coccidiosis. In contrast, others sought a solution to a technical problem and emphasized its use in the technological field of formulations and products, adding value to pharmaceutical technology. Therefore, some of the patents were highlighted (Table 4) because they correlate with the objective of this work. For example, Wang’s research group has recently obtained patents that introduce nanoscale dosage forms to improve the biopharmaceutical properties of DQ to enhance the distribution of the drug. It is worth noting that some patents have emerged from previously published articles.

## 8. Perspectives

**Figure 6:**
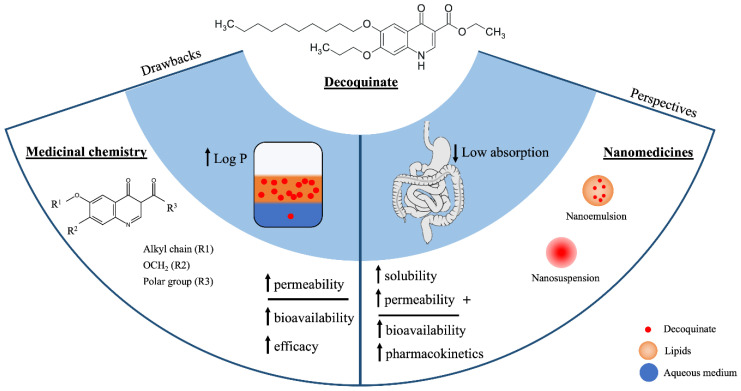
Drawbacks of and perspectives on decoquinate research.

Due to the low solubility of the drug in aqueous fluids, this molecule needs to be optimized for human use, or its formulation rationalized to increase its apparent solubility [26]. The synthesis of new DQ derivatives aims to improve its biopharmaceutical properties through modifications that alter important aspects, such as the physicochemical properties and the reduction in its easy metabolization that occur due to the intrinsic properties of the molecule. For this reason, the study of derivatives is valuable for increasing the possibility of addressing multiple occurrences. However, once new molecules are involved, the time to market may be longer than for pharmaceutical technology approaches where the molecule is not chemically altered, and therefore, some of the safety/toxicology studies do not need to be repeated (Figure 6).

An alternative to circumvent the problem of insolubility is to explore formulation methods. The application of pharmaceutical nanotechnology is an alternative to overcome these inherent problems of DQ by using extrinsic factors of the molecule. In this context, pharmacokinetic studies have shown that by increasing the solubility of DQ in aqueous fluids through the preparation of micro- and nanosuspensions, relevant improvements in the dissolution rate, permeability, and consequently oral bioavailability, were observed in rats [52,79]. In addition, lipid-based systems such as liposomes, niosomes, and transferosomes have also been described. However, the stability of these systems is closely related to the balance of lipid bilayer interactions, which are easily disturbed by DQ once the molecule is anchored in this region of the nanocarrier [12]. Therefore, systems based on lower structural complexity—such as nanoemulsions and microemulsions—that improve biopharmaceutical properties might provide better results.

In recent years, some products already on the market have been optimized by solid or liquid dispersions, including colloidal dispersions. There are also other systems of liquid crystals that have the fluidity of liquids but retain the structure and molecular arrangement of solids, especially lyotropic systems that favor the formation of micelles [93,94,95]. These methods aim to achieve increased solubility, stability, and altered bioavailability. In addition, they serve as drug delivery systems that would also be useful for improving the biopharmaceutical properties of DQ.

## 9. Conclusions

DQ is an effective and established antimicrobial drug in veterinary medicine, both in prophylaxis and long-term treatment to control parasitic infections. Preclinical studies have shown that DQ has a broad spectrum of antimicrobial activity and is suitable not only for the treatment of infections in veterinary medicine but also for treating humans and even pathogens that affect both animals and humans, such as mycobacteria, toxoplasmosis, and schistosomiasis. Several limitations remain to be overcome in the treatment of humans with DQ, such as high lipophilicity and a lack of information on human administration, biodistribution, and pharmacokinetic profile. Nevertheless, in the literature, most studies conducted on the biodistribution and pharmacokinetics of DQ in rodent models suggest a potential antimicrobial therapy. New pharmaceutical formulations need to be explored to optimize biopharmaceutical and pharmacokinetic properties, and in this branch of research, liposomes, nanosuspensions, and microemulsions may improve the chances of DQ use in humans. In addition, the chemical derivatization of DQ is also a route to drug development. A combination of chemical derivatization and pharmaceutical nanotechnology will most likely overcome the limitations of DQ and provide a potential therapeutic alternative for various infections.

## Figures and Tables

**Table 2:** In vivo pharmacokinetic parameters of micro- and nanostructured DQ after oral administration (80 mg/Kg) to male ICR mice [79] and intramuscular administration (120 mg/kg) to male ICR mice [49].

Dose	80 mg/Kg [79]		120 mg/Kg [49]	
Parameters	Microsuspension (36.88 µm) *	Nanosuspension (0.39 µm) *	Microsuspension (8.31 μm) *	Nanosuspension (0.43 μm) *
AUC inf. (ng·h/mL or g)	51.4 ± 6.2	795.5 ± 36.19	18,311 ± 926	10,385 ± 405
C_max_ (ng/mL or g)	6.1 ± 0.7	25.3 ± 2.9	45.35 ± 8.09	36.58 ± 3.56
t_1/2_ elimination (β, h)	8.17 ± 2.59	23.93 ± 14.20	1444.90 ± 118.56	751.01 ± 28.72
CL/F (L/h/kg)	313.6 ± 37.6	117.8 ± 58.9	6.57 ± 0.32	11.56 ± 0.45
BA (Relative) (%)	6.77	100	-	-

* Average particle diameter.

**Table 3:** Pharmacokinetic evaluation in male C57BL mice using doses of DQ derivatives of 20 mg/kg (oral) (*n* = 3) and 5 mg/kg (IV) (*n* = 3) [17].

Parameters	IV			Oral		
	RMB041	RMB043	RMB073	RMB041	RMB43	RMB073
AUC (min. µmol/L)	29,250.4 ± 309.0	10,068.4 ± 127.8	15,940.0 ± 400	25,012.2 ± 108.0	8915.7 ± 1017.0	3771.0 ± 296.0
C_max_ (µM)	-	-	-	5.4 ± 0.4	5.6 ± 1.4	2.0 ± 0.3
t_1/2_ (h)	62.3 ± 6.7	8.6 ± 0.4	15.3 ± 3.2	23.4 ± 2.5	6.2 ± 0.8	11.6 ± 1.3
CL tot (mL/h/kg)	23.1 ± 0.3	70.5 ± 4.2	34.5 ± 1.3	-	-	-
Vd (L/kg)	1.2 ± 0.03	4.6 ± 1.6	3.9 ± 0.5	-	-	-
Bioavailability	-	-	-	21.4 ± 1.0	22.1 ± 2.2	5.9 ± 1.3

**Table 4:** Summary of patents related to the use of pharmaceutical technology approaches to improve the biopharmaceutical properties of DQ.

Information	PATENTS								
	US5200418	US20170360708A1	WO2012/174121A2	CN110898096 (A)	CN110585134 (A)	CN109620805 (A)	WO2019217957	US20210059946	US20210077480
Publication date	4/6/93	12/21/17	12/20/12	3/24/20	12/20/19	4/16/19	11/14/19	03/04/21	03/18/21
Inventors	Redman et al. [84]	Wang et al. [85]	Pogany et al. [86]	Chen et al. [87]	Zeng et al. [88]	Yuan et al. [89]	Huang et al. [90]	Wang et al. [91]	Wang et al. [92]
Title	Cryptosporidiosis amelioration	Solid dispersion of decoquinate, a preparation process, and its application	Decoquinate prodrugs	Decoquinate microcapsules and preparation method thereof	Decoquinate liposome and preparation method and application thereof	Preparing method of decoquinate dry suspension	Quinoline compounds and their preparation and use as antimalarial agents	Intramuscular depot of decoquinate compositions and method of prophylaxis and treatment thereof	Nanoparticle formulations of decoquinate in the form of solid solution
Benefited	The Ohio State University Columbus, OSU (EUA).	Guangzhou Cas Lamvac Biotech Co., Ltd. (China).	University of Kansas (EUA).	Foshan Standart Bio Tech Co., Ltd. (China).	Guangzhou Lamvac Pharmaceutical tech Co., Ltd. (China).	Guangzhou Wens Dahuanong Biotechnology Co., Ltd. (EUA).	The Henry M. Jackson Foundation For The Advancement Of Military Medicine Inc. (EUA).	Bluelight Pharmatech Co., Ltd. (China).	Bluelight Pharmatech Co., Ltd. (China).
